# Slow Recovery from Inbreeding Depression Generated by the Complex Genetic Architecture of Segregating Deleterious Mutations

**DOI:** 10.1093/molbev/msab330

**Published:** 2021-11-17

**Authors:** Paula E Adams, Anna B Crist, Ellen M Young, John H Willis, Patrick C Phillips, Janna L Fierst

**Affiliations:** 1 Department of Biological Sciences, University of Alabama, Tuscaloosa, AL, USA; 2 Department of Genomes and Genetics, Institut Pasteur, Paris, France; 3 Institute of Ecology and Evolution, University of Oregon, Eugene, OR, USA

**Keywords:** conservation genetics, genomics, inbreeding depression, nematode

## Abstract

The deleterious effects of inbreeding have been of extreme importance to evolutionary biology, but it has been difficult to characterize the complex interactions between genetic constraints and selection that lead to fitness loss and recovery after inbreeding. Haploid organisms and selfing organisms like the nematode *Caenorhabditis elegans* are capable of rapid recovery from the fixation of novel deleterious mutation; however, the potential for recovery and genomic consequences of inbreeding in diploid, outcrossing organisms are not well understood. We sought to answer two questions: 1) Can a diploid, outcrossing population recover from inbreeding via standing genetic variation and new mutation? and 2) How does allelic diversity change during recovery? We inbred *C. remanei*, an outcrossing relative of *C. elegans*, through brother-sister mating for 30 generations followed by recovery at large population size. Inbreeding reduced fitness but, surprisingly, recovery from inbreeding at large populations sizes generated only very moderate fitness recovery after 300 generations. We found that 65% of ancestral single nucleotide polymorphisms (SNPs) were fixed in the inbred population, far fewer than the theoretical expectation of ∼99%. Under recovery, 36 SNPs across 30 genes involved in alimentary, muscular, nervous, and reproductive systems changed reproducibly across replicates, indicating that strong selection for fitness recovery does exist. Our results indicate that recovery from inbreeding depression via standing genetic variation and mutation is likely to be constrained by the large number of segregating deleterious variants present in natural populations, limiting the capacity for recovery of small populations.

## Introduction

“The evil effects of close interbreeding” (Darwin 1896) have been of interest to biologists since the 1800s (Bemiss 1858). Mating between closely related individuals can result in inbreeding depression, a loss of fitness (Charlesworth and Charlesworth 1987) that can affect endangered or isolated species (Kardos et al. 2016) and lead to the eventual extinction of small populations (Hedrick and Garcia-Dorado 2016). However, despite a developed understanding of the significance of inbreeding depression, identifying specific alleles underlying the reduction in fitness has remained challenging (Hedrick and Garcia-Dorado 2016). From a conservation point of view, we know even less about the likelihood that populations with a history of inbreeding can recover fitness (Hedrick and Kalinowski 2000).

During inbreeding, deleterious alleles have three potential trajectories. They may be: 1) purged after exposure to stronger selection, 2) fixed as large regions of the genome become homozygous, or 3) maintained as heterozygous sites in the genome (Charlesworth and Charlesworth 1987; Charlesworth and Willis 2009). Purging deleterious alleles is often key to survival in inbreeding populations, while fixation of these alleles shifts the population from its current fitness optimum and results in genetic load (Hedrick 1994; Robinson et al. 2018, 2019). However, some species maintain higher levels of genetic diversity even after extensive inbreeding (Eriksson et al. 1976; Latter 1998; Roessler et al. 2019; Smith et al. 2019; Waller 2021). If fixing the region in either direction causes a severe reduction in fitness, then the region may remain heterozygous (Charlesworth and Willis 2009). The region may be truly overdominant (heterosis) or “pseudo-overdominant,” where deleterious alleles are linked in repulsion. These regions will remain heterozygous until the deleterious alleles can be broken up by recombination (Charlesworth and Willis 2009; Waller 2021). Pseudo-overdominance has been linked to the inability to purge deleterious mutations leading to worsening inbreeding depression (Latter 1998; Hedrick et al. 2016; Seymour et al. 2016; Roessler et al. 2019). For example, apparent overdominance in maize was later identified as pseudo-overdominance with repulsion linkage hiding deleterious alleles (Crow 1999). Heterosis for height in sorghum was also found to be caused by two quantitative trait loci linked in repulsion (pseudo-overdominance; Li et al. 2015). The specific genetic architecture of inbreeding depression has implications for fitness recovery as populations unable to purge deleterious alleles may have a longer and more complex road to recovery.

Inbreeding depression in most populations is likely generated by the accumulation of segregating mutations over a long period of time and potentially at a large number of loci. Thus, while effects like those observed in mutation accumulation studies (Charlesworth et al. 1993) are the ultimate source of inbreeding depression in natural populations, they may not reflect the long-term segregating effects of mutations that have been filtered through population-level processes of natural selection, genetic drift and genomic linkage. Inbreeding assays of natural isolates have shown minimal fitness loss in the self-fertilizing *Caenorhabditiselegans* but very severe fitness loss and up to ∼90% extinction in inbred lines of the obligate outcrossing *C*. *remanei* (Dolgin et al. 2007), with the difference almost certainly driven by the amount of genetic load remaining after selection under these two mating systems (Lande and Schemske 1985). Thus, while we expect that inbred populations *can* recover after the fixation of deleterious mutations (Estes and Lynch 2003; Denver et al. 2010; Estes et al. 2011), whether they *will* recover is an open question that may be dependent upon the ability to purge genetic load during inbreeding. Previous research has indicated that species with high genetic load may not be capable of evolutionary rescue—adaptation after negative environmental changes (Stewart et al. 2017)—and highly inbred populations may further deteriorate with genetic rescue from a population with high genetic load (Robinson et al. 2019; Kyriazis et al. 2021). However, recent debate about maximizing genetic diversity versus minimizing deleterious alleles has left the question open for what is best for each species or subpopulation, especially those with high genetic load (Robinson et al. 2018, 2019; Ralls et al. 2020; Scott et al. 2020; Kyriazis et al. 2021).

Here, we use whole-genome sequencing in *C. remanei* to study allelic changes that accompany fitness loss through inbreeding and track alleles in three replicate populations as they recover from inbreeding. Analyzing the first phase of inbreeding allows us to quantify how many sites were fixed during this process, as well as how many sites displayed resistance to inbreeding. Analyzing the second phase of recovery allows us to observe parallel genomic changes across lines that recovered to large population sizes. Our results show that, in contrast to expectations generated from haploid and self-fertile organisms, fitness recovery from inbreeding may not be easily accomplished in *C. remanei* because of the scope and scale of segregating deleterious genetic variation within male–female populations.

## Results

### Fecundity Decreased and Longevity Increased with Inbreeding

**Fig. 1.**
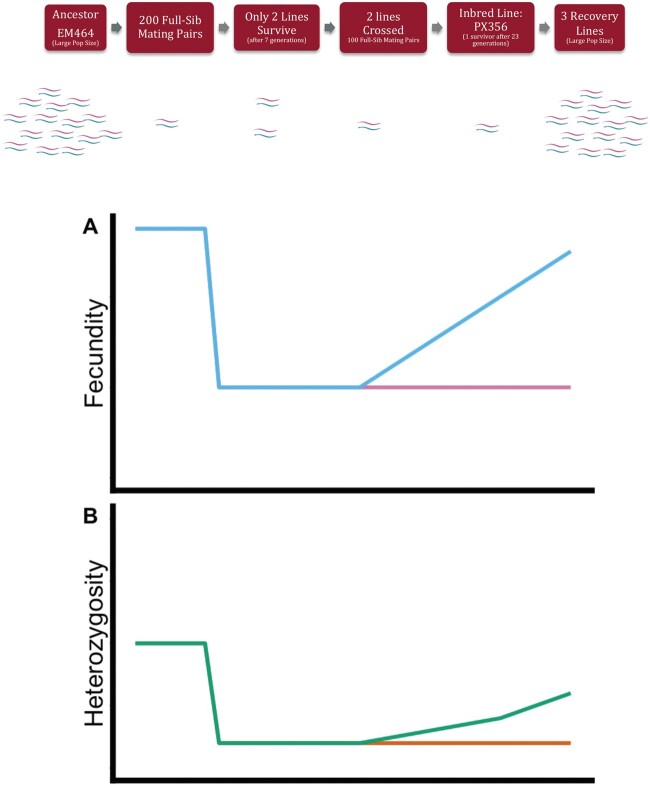
(*A*) The inbreeding and recovery scheme used to create the Inbred line from the Ancestral strain of *C. remanei*. Two hundred plates with full-sibling mating pairs were kept through seven generations until only two remained alive. Those 2 lines were allowed to expand for 20 generations then crossed to create 100 full-sib mating pairs. These lines were transferred for 23 generations until only one line, the Inbred PX356, was left alive. Offspring of the Inbred line were allowed to reproduce at large population size in 3 replicate Recovery lines for 300 generations. (*B*) We measured fecundity and heterozygosity in Ancestor, Inbred, and Recovery to analyze the potential of an outcrossing, diploid population to recover from inbreeding.

The ancestral *C. remanei* strain EM464 (hereafter referred to as “Ancestor”) was obtained from the Caenorhabditis Genetics Center, University of Minnesota, Minneapolis, MN (Baird et al. 1994; Sudhaus and Kiontke 1996). Full sibling males and females were crossed for >23 generations to create strain PX356 (hereafter referred to as “Inbred”; breeding scheme shown in fig. 1; Fierst et al. 2015). About 99% of inbred lines went extinct quickly during full-sib mating, indicating that just surviving inbreeding in *C. remanei* is the exception, not the rule. Three lines (hereafter referred to as “Recovery”) were independently established from the Inbred population and allowed to recover from inbreeding via random mating at large population size (∼10,000 worms).

**Fig. 2.**
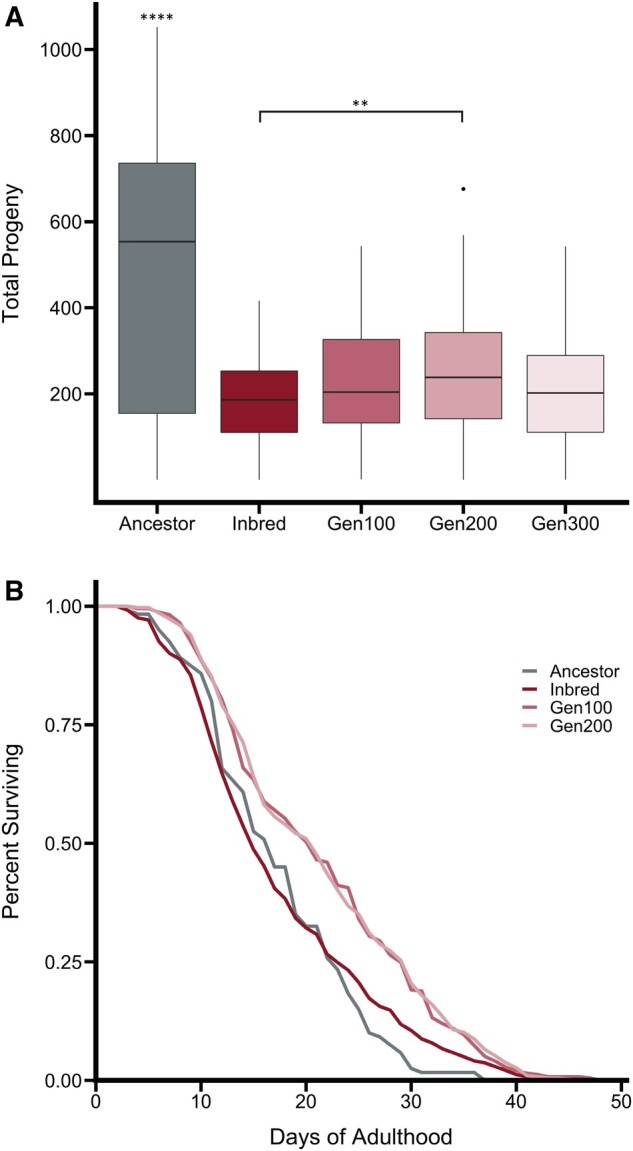
The phentypic effects of inbreeding included (*A*) a decrease in the mean reproductive output that was not recovered after 300 generations of breeding at large population sizes. There was (*B*) no influence of inbreeding on longevity but the Recovery lines evolved an increase in longevity when compared with the Ancestral and Inbred lines.

After inbreeding and recovery, fecundity, and longevity assays were conducted on population samples. The Inbred line was included in each experiment as a control. The mean cumulative progeny per individual for the Ancestor was 494 (SD 321) and decreased to 182 (SD 103) in the Inbred line, a 63% reduction (fig. 2*A*; table 1; supplementary table 1, Supplementary Material online; *P*-adj 1.8 e^−36^). Total progeny increased by 34% to 244 (SD 145) after 200 generations of recovery (*P*-adj 8.3 e^−3^; Table 1). However, by 300 generations the mean progeny decreased to 207 (SD 129) (fig. 2*A*). The mean lifespan in the Recovery lines was 4 days longer than that of the Ancestor, and the oldest individual in the Recovery lines lived 12 days longer than the longest living Ancestor (fig. 2*B*).

**Table 1.** Paired *t-*Test Results for Average Fecundity.

Group 1	Group 2	*P*-value	*P*.signif	*P*.adj (Bonferroni)	*P*.adj.sigif
Ancestor	Inbred	1.80E−37	****	1.80E−36	****
Ancestor	Gen100	3.15E−27	****	3.15E−26	****
Ancestor	Gen200	1.10E−25	****	1.10E−24	****
Ancestor	Gen300	3.57E−30	****	3.57E−29	****
Inbred	Gen100	0.0369	*	0.369	ns
Inbred	Gen200	0.00083	***	0.0083	**
Inbred	Gen300	0.211	ns	1	ns
Gen100	Gen200	0.294	ns	1	ns
Gen100	Gen300	0.429	ns	1	ns
Gen200	Gen300	0.0591	ns	0.591	ns

Note.—Ancestor is significantly different from all inbred and recovery lines. Inbred line is significantly different from Recovery Generation 200.

(*P* < 0.05 = *; *P* < 0.01 = **; *P* < 0.001 = ***; *P* < 0.0001 = ****).

**Fig. 3.**
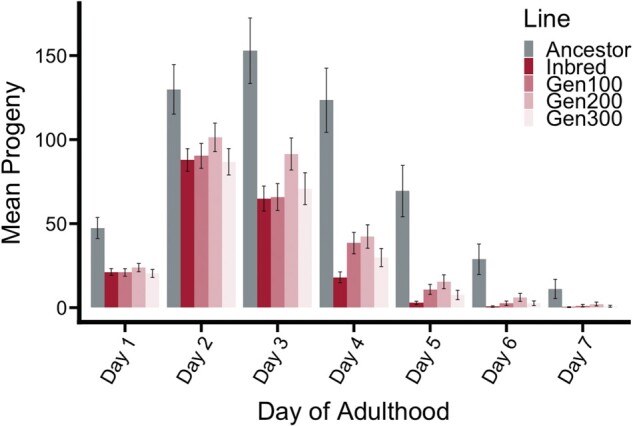
Mean progeny by day of adulthood.

Age-specific fecundity differed among lines (fig. 3). The Inbred line completed 90% of its egg laying within the first 3 days of reproduction and 100% of its egg laying within 5 days. In comparison, the Ancestor completed 52% of its egg laying within the first 3 days of reproduction and continued egg laying at a low rate for the 7 day assay period. The Recovery lines completed 76–81% of their egg laying within the first 3 days and continued egg laying at decreasing rates for 7 days.

### Allelic Diversity Declined Less Than Expected

In order to study the change in allele frequency during inbreeding we sequenced populations prior to inbreeding (Ancestor “EM464”), after inbreeding (Inbred “PX356”), 3 Recovery replicates at 100 generations, and 3 Recovery replicates at 200 generations. We aligned DNA sequences to the assembled *C. remanei* PX356 reference and used the MAPGD *pool* function to estimate allele frequencies (Lynch et al. 2014; Ackerman et al. 2017) and identify single nucleotide polymorphisms (SNPs). The *C. remanei* genome is a draft reference assembled from paired-end Illumina libraries and ∼5.5% shorter than the estimated genome size of 126 Mb (Fierst et al. 2015; Teterina et al. 2020). We implemented a set of rigorous filters on our genotype estimations to avoid issues from repetitive and complex regions potentially missing from the *C. remanei* PX356 reference (described in detail in Materials and Methods). After filtering, 150,348 SNPs remained segregating across the populations (sequencing coverage is given in supplementary table 2, Supplementary Material online).

**Fig. 4.**
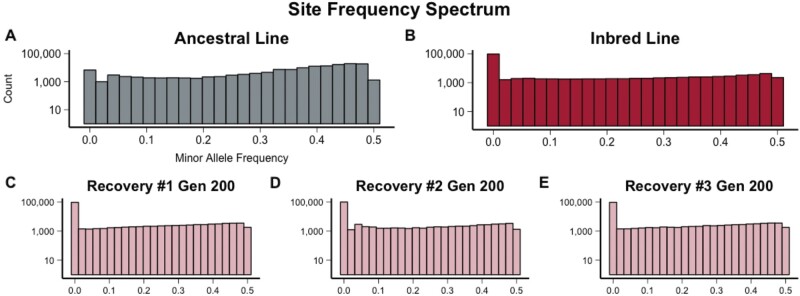
The minor allele SFS showed (*A*) a majority of sites with minor allele frequencies 30–50% in the Ancestral line. This was altered through inbreeding and (*B*) the increase in fixation resulted in 98,940 fixed sites in the Inbred Line. Despite the intensity of inbreeding 48,490 sites still had segregating minor alleles. Recovery lines 1 (*C*), 2 (*D*), and 3 (*E*) had 9,394 shared sites retain fixation from the inbred line and 2,261 shared segregating minor alleles.

The number of polymorphic sites was reduced during inbreeding (fig. 4*A*–*E*). Of the 150,348 segregating sites observed, 139,658 (93%) were variable in the Ancestor and 51,408 (34%) were variable in the Inbred line. To compare the amount of fixation to expected values, we used the formula ∼1.17 *h_0_*(0.809)^*t*^ where *h_0_* is initial heterozygosity and *t* is the number of generations (Nagylaki 1992). With >23 inbreeding generations we expect no more than ∼0.89% of the initial heterozygosity to remain. Thus, of 139,658 segregating sites in the Ancestral line, we would expect 1,248 SNPs to remain after inbreeding. The observed value of 51,408 SNPs in the surviving inbred line suggests regions that escaped fixation (Barrière et al. 2009). The Recovery lines had an average of 45,853 SNPs (∼30% of those identified in the Ancestor) in generation 100 and 50,593 SNPs (∼34% of those identified in the Ancestor) in generation 200. This is likely an underestimate of true segregating diversity in recovery due to our strict coverage filters and rigorous requirements to define segregating SNPs but it demonstrates that little genetic variation was regained or generated in recovery.

### F_ST_ Demonstrated a Bimodal Response to Inbreeding

**Fig. 5.**
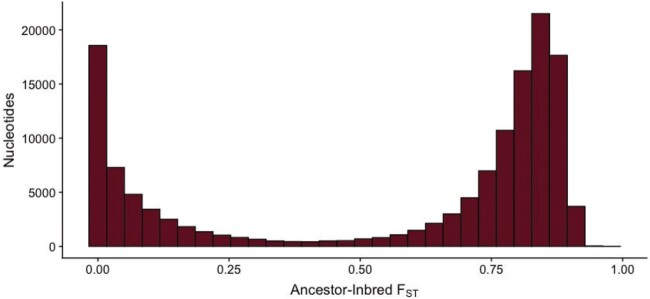
The frequency distribution of F_ST_ calculated between Ancestor and Inbred lines shows that there is a bimodal response to inbreeding with many sites showing no divergence in allele frequency (i.e., F_ST_ ∼0) between Ancestor and Inbred lines and other sites showing high divergence in allele frequency in response to inbreeding (i.e., F_ST_ > 0.6).

Given the high rate of segregating sites remaining after inbreeding, we sought to characterize the extent of allelic change between the Ancestor and Inbred lines. The mean per-site F_ST_ between Ancestor and Inbred was 0.5 and the distribution was strongly bimodal (fig. 5). The bimodal distribution shows that while some sites were quickly fixed during inbreeding, other sites remained nearly the same after inbreeding. Roughly 30% of the SNPs in this comparison (74,505) had F_ST_ < 0.1 indicating little allelic divergence between the Ancestor and Inbred at these sites. In contrast, ∼60% of the segregating sites had substantial F_ST_ > 0.5 between the Ancestor and Inbred.

### Heterozygosity was Retained Throughout the Genome

**Fig. 6.**
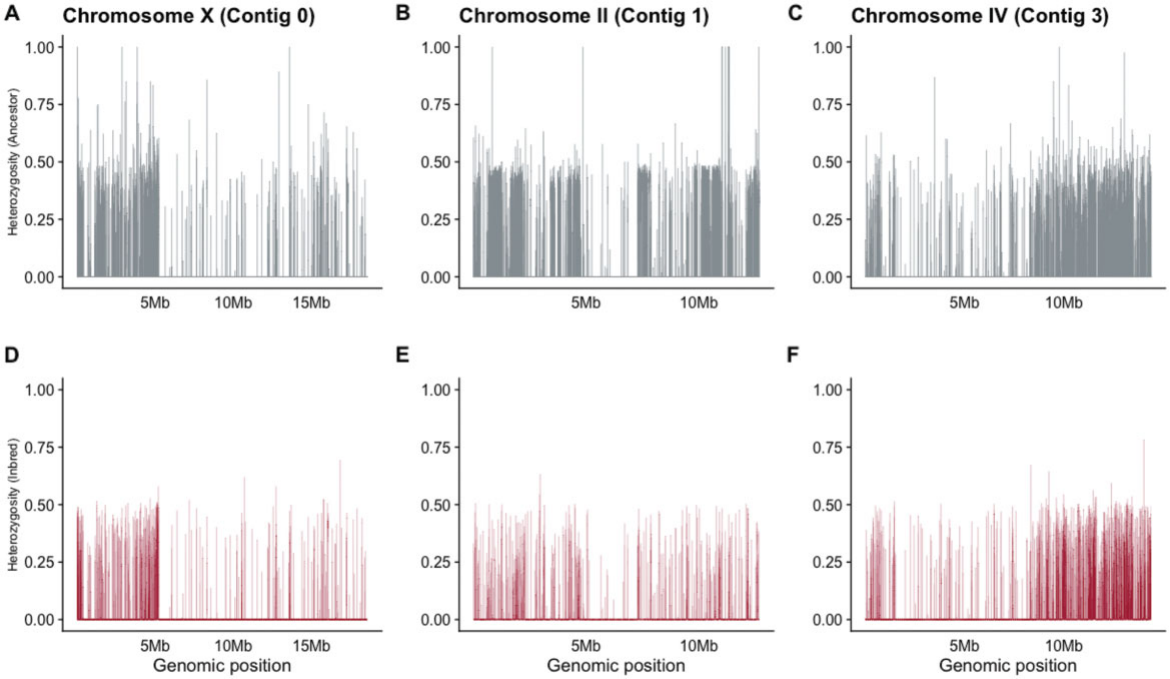
Average heterozygosity in 1 kb blocks across the three largest linkage groups, corresponding to (*A*) Chromosomes X, (*B*) II and (*C*) IV) show that polymorphism in the Ancestor line was decreased through inbreeding but regions of segregating variation remained in the Inbred line (*D–F*). Residual segregating polymorphisms are not evenly distributed along chromsomes and there are distinct regions of Chromosome X and IV that retain polymorphism in the Inbred line.

To identify genomic regions of high heterozygosity, we averaged minor allele frequencies across 1 kb windows for each population. We defined a fixed region as a contiguous portion of the genome 1 kb or greater in length where minor allele frequency did not exceed 3%. Fixed regions were smaller in the Ancestor and larger in the Inbred population, however, high heterozygosity was retained across all contigs (fig. 6*A*–*F*). Chromosome X showed little change in heterozygosity after inbreeding (fig. 6*A* and *D*) while Chromosome II showed the greatest decrease in heterozygosity after inbreeding (fig. 6*B* and *E*). Roughly one half of Chromosome IV decreased in heterozygosity after inbreeding (fig. 6*C* and *F*) while the second half retained heterozygosity through both inbreeding and recovery. Fixed regions increased in size and frequency in the Inbred line as compared with the Ancestor, however, large regions (>50 kb) of fixation were extremely rare (supplementary fig. 1, Supplementary Material online). Heterozygosity is not evenly distributed along the chromosomes in *C. remanei;* similar to the pattern of punctuated divergent regions in *C. elegans* (Lee et al. 2021). Recovery Lines showed similar heterozygosity distributions to the Inbred Line (supplementary fig. 2, Supplementary Material online).

### Allele Frequency Trajectories Differed between the X Chromosome and Autosomes

**Fig. 7.**
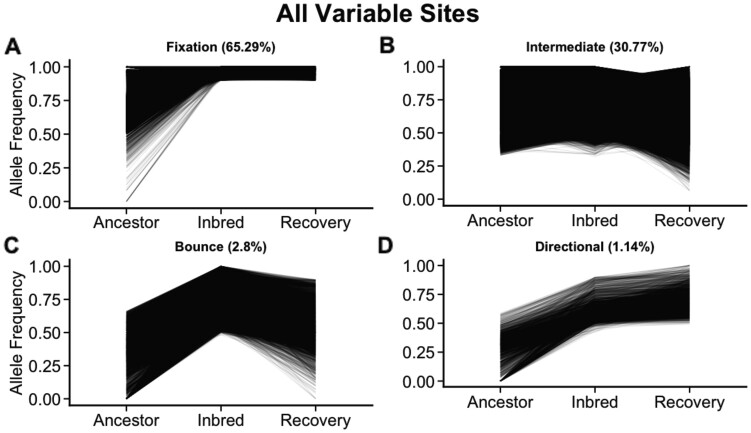
Across the entire genome allele frequency trajectories demonstrate that a majority of sites were either (*A*) fixed through inbreeding and remained fixed during recovery or (*B*) maintained intermediate allelic frequencies through both inbreeding and recovery. A minority of sites demonstrated allelic frequencies that were (*C*) low in the Ancestral line, raised through inbreeding and lowered again in the Recovery lines; (*D*) rose in frequency through inbreeding and rose further in the Recovery lines.

To visualize patterns of allele frequency change during inbreeding, we divided sites into four trajectories: Fixation, Intermediate, Bounce, and Directional. Fixation sites were those that were variable in the Ancestor and then fixed in the Inbred line (<5% allele frequency in the population). Intermediate sites were those that were variable in the Ancestor and remained variable in the Inbred line (<50% allele frequency change throughout inbreeding and recovery). The remaining segregating sites that changed >50% during inbreeding and recovery were characterized by their behavior during recovery into two groups: Bounce and Directional. Bounce sites were those that were segregating at high or low frequencies in the Ancestor, changed >50% in the Inbred line, and reverted to original frequency in Recovery. And finally, Directional sites were those with low frequency in the Ancestor and increased in the Inbred and Recovery populations. Of the 150,348 SNPs, 98,160 (65.29%) were segregated in the Ancestor and went to “Fixation” in the Inbred (fig. 7*A*). An additional 46,267 (30.77%) were maintained at “Intermediate” frequencies in Inbred and Recovery (fig. 7*B*). A small proportion of sites (4,211; 2.8% of the total variation) “Bounced,” back to low frequency in Recovery (fig. 7*C*). A small minority of sites (1,707; 1.14% of the total set) changed allele frequency in a Directional pattern (fig. 6*D*).

**Fig. 8.**
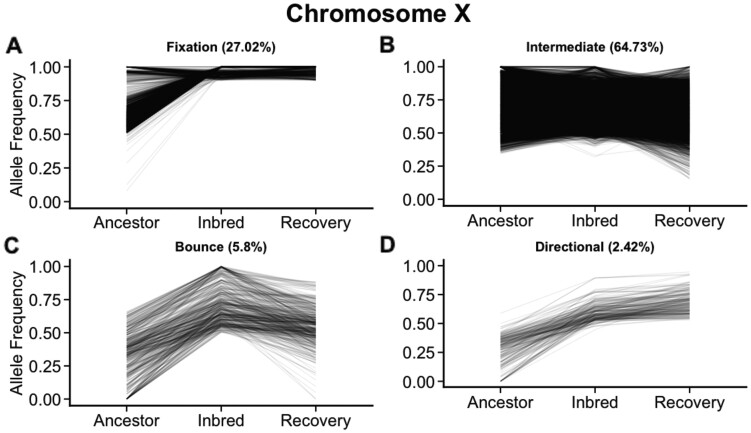
Variants on the X Chromosome were less likely to (*A*) fix through inbreeding and (*B*) more likely to remain at intermediate frequency through inbreeding and recovery. A small proportion of sites on the X chromosome also showed parallel patterns of variable allele frequencies (*C*–*D*).

Segregating sites on the X chromosome exhibited different patterns (fig. 8) with only 2,137 (27.02%) of the segregating sites showing a “Fixation” pattern and 5,119 (64.73%) of sites segregating as “Intermediate.” The remaining 652 (8.47%) sites showed Bounce and Directional patterns. In total 7,908 segregating sites (5.3% of the total set) resided on the X chromosome. The X chromosome is 18.6 Mb and roughly 16% of the assembled 118.5 Mb *C. remanei* genome. While the density of SNPs on the X Chromosome was lower in the Ancestor, these sites displayed high fixation resistance during inbreeding.

### Estimated Recombination Rates Decreased in the Inbred Line

**Fig. 9.**
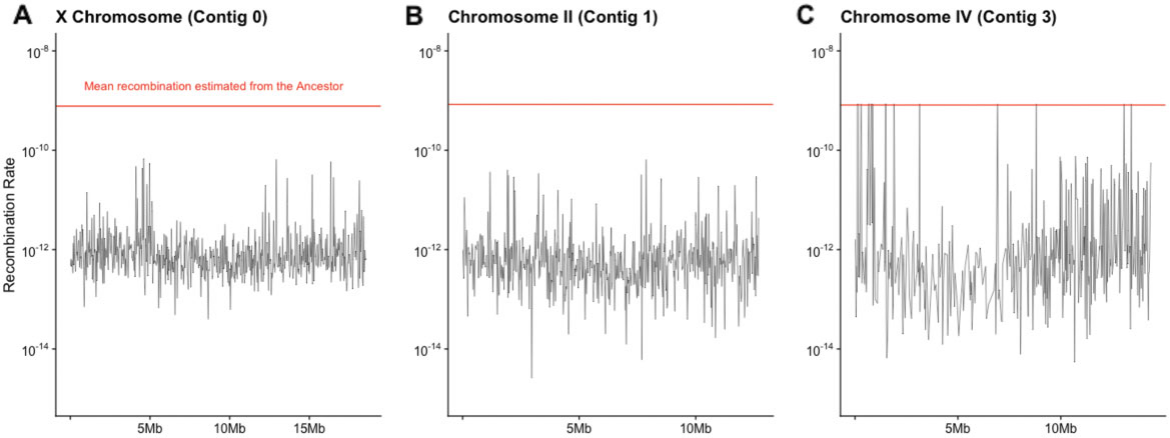
Recombination rates in the Inbred line (black) across the three largest linkage groups: (*A*) the X Chromosome (contig0), (*B*), Chromosome II (Contig1), and (*C*) Chromosome IV (Contig3). Average recombination rate for the ancestral line on each chromosome is shown as a red line in each plot.

If punctuated regions of elevated heterozygosity are maintained due to deleterious alleles linked in repulsion (pseudo-overdominance), these regions could also show decreased rates of recombination (Waller 2021). To further investigate the elevated heterozygosity maintained in our inbred line, we estimated the recombination landscape using the pooled-sequencing pipeline in the software package ReLERNN (Adrion et al. 2020). We were initially unable to get fine-scaled recombination landscapes with our filtered, high-confidence SNPs. After expanding to all possible polymorphic sites, we obtained fine-scaled recombination estimates for the Inbred line and coarse-scaled recombination rates for the Ancestor (fig. 9*A*–*C*). The average recombination rate for the Ancestor was comparable to previously reported averages in *C. elegans* (Rockman and Kruglyak 2009). In comparison, the Inbred line had severely reduced recombination rates across the genome. Due to the reduced recombination rate and patterns of retained heterozygosity, we used the Haplovalidate software (Franssen et al. 2017; Otte and Schlotterer 2021) to identify haplotypes in the Inbred and Recovery populations but the haplotypes were not statistically significant.

### Effective Population Size Did Not Recover

We used the software package poolSeq (Taus et al. 2017) to estimate the effective population size in the Inbred and Recovery lines. The effective population size of wild-collected *C. remanei* has been previously estimated to be ∼1,000,000 (Cutter et al. 2006). The effective population size for the Inbred line was estimated as 28 (supplementary table 3, Supplementary Material online). The three Recovery lines had a mean effective population size of 90 after 100 generations and 148 after 200 generations (supplementary table 3, Supplementary Material online).

### Selection Scans Identified Significant Parallel Changes in Recovery

In order to detect any possible genetic recovery from inbreeding, we used two methods to identify significant, parallel, allele frequency changes across Recovery lines. First, we fit a generalized linear model (GLM) with quasibinomial error distribution to the allele frequency changes across the Inbred line, generation 100 Recovery, and generation 200 Recovery according to Wiberg et al. (2017) recommendation for best practices with pooled sequencing data. Second, we performed a Cochran–Mantel–Haenszel (CMH) test to analyze parallel changes in allele frequencies between the Inbred and Recovery lines at generation 100 and 200 with the software package PoPoolation2 (Kofler et al. 2011). All sites that were significant in the quasibinomial-GLM analyses were also significant with the CMH test, and we retained all significant sites for analysis.

The quasibinomial-GLM revealed 102 SNPs with significant parallel changes across the three Recovery lines (q-value < 0.05). Of these 102 SNPs, 36 were contained within 30 genes. Genomic locations, log q-values from the quasibinomial-GLM, and snpEff annotation (Cingolani et al. 2012) for these genes are given in table 2. InterProScan protein domain annotations (Jones et al. 2014) and *Caenorhabditis* orthologs (identified with the OrthoFinder software package; Emms and Kelly 2015) for these genes are listed where available. Several genes had no identifiable domain annotations or orthologous proteins in other species. Seventeen of the sites were characterized as Intron Variants with potential modifier effects. Nine sites were characterized as Synonymous Variants of low effect. Seven sites were characterized as Missense Variants of moderate impact. One site was a Splice Region intron variant. Only one site was characterized as high impact and was predicted to cause a Loss of Stop impact.

**Table 2.** Genomic Location, log q-Values from the Quasibinomial-GLM, and Gene Name for Each of the Genes with Significant SNPs in Our Allele Frequency Scans.

Location	GLM Results		Gene, Ortholog, and Protein Information		
	log(q-value)	Slope	Gene	*C. elegans* or other *Caenorhabditis* orthologous protein	InterProScan and snpEff Annotations
Contig: position					
0: 753186; 753191	2.3; 2.3	25.5; 25.4	FL81_00147	—	Intron Variants
0: 5208517	2.3	25.0	FL81_01105	—	Synonymous Variant
0: 10099911	2.3	25.8	FL81_02098	**F47B7.2**; ortholog of human *QSOX1* and *QSOX2*; predicted to have thiol oxidase activity; expressed in the head and alimentary, epithelial, muscular and reproductive systems.	IPR007248 Mpv17/PMP22; Intron Variant, Splice Region Variant
0: 10539106	2.1	1.8	FL81_02186	**C07A12.7**; ortholog of human *TOM1* and *TOM1L2*; human *TOM1L2* exhibits clathrin and protein kinase binding activity.	Synonymous Variant
0: 17433630; 17436635; 17436636	2.3; 2.3; 2.3	24.4; 24.4; 24.4	FL81_03749	**C18B12.6**; ortholog of human *ERGIC2*; expressed in tail neurons, the anal depressor and sphincter muscles, the gon_herm_dtc_A, and the gon_herm_dtc_P; predicted to encode an Endoplasmic reticulum vesicle transporter, C-terminal domain.	Intron Variants
1: 1473060	1.6	−3.2	FL81_06934	—	Intron Variant
1: 1896140	2.3	26.5	FL81_07024	*C. nigoni* Cnig_chr_II.g7686	IPR021942 Protein of unknown function DUF3557; Intron Variant
1: 10958032	2.3	25.4	FL81_08858	**K10G6.4**; expressed in the head, the nervous system, and the sensillum.	Intron Variant
3: 9671404	2.3	25.4	FL81_06059	—	IPR019421 7TM GPCR, serpentine receptor class d (Srd); Intron Variant
3: 13012323	2.3	25.3	FL81_06442	**R05A10.2;** enriched in the PLM, amphid sheath cell, hypodermis, and intestine; affected by several genes including *daf-2*, *elt-2*, and *eat-2*.	Intron Variant
5: 19922641	2.3	25.4	FL81_10201	Part of a co-orthologous group with ten C*. nigoni* proteins	Stop Lost, Splice Region Variant—High Impact
7: 499146	2.3	3.7	FL81_10375	—	IPR001810 F-box domain; IPR002900 Domain of unknown function DUF38, *Caenorhabditis* species; Intron Variant
7: 1662659	2.3	24.8	FL81_10646	*C. angaria* Cang_2012_03_13_05027.g19294	Missense Variant
7: 1772080	2.3	26.2	FL81_10653	*C. brenneri* CBN03163	Synonymous Variant
7: 1905299	1.9	−1.6	FL81_10668	**F45D11.9** *fbxc-42* and **R07C3.9***fbxc-31*; both predicted to encode a Protein of unknown function DUF3557 domain.	Missense Variant
8: 807030	1.5	2.5	FL81_12328	—	IPR021942 Protein of unknown function DUF3557; Missense variant
8: 906126	2.3	25.9	FL81_12343	—	IPR021109 Aspartic peptidase domain; Synonymous Variant
10: 910662	2.3	−2.2	FL81_11390	**R03D7.4** *tceb-3*; ortholog of human *ELOA* (elongin A), *ELOA2* (elongin A2), and *ELOA3D* (elongin A3 D), involved in transcription elongation from RNA polymerase II promoter; localizes to the transcription elongation factor complex; expressed in the alimentary, muscular, nervous and reproductive systems.	IPR001810 F-box domain; Intron Variant
10: 1259520	2.3	25.6	FL81_11446	—	IPR001810 F-box domain; IPR012885 F-box domain, type 2; Intron Variant
74a: 265885	1.5	0.99	FL81_17225	**F21D12.3**; expressed in motor neurons and the body wall musculature; predicted to encode an Amino acid transporter, transmembrane domain.	Missense Variant
93: 105170	1.4	1.0	FL81_17378	—	IPR019420 7TM GPCR, serpentine receptor class bc (Srbc); Synonymous Variant
93: 132161	2.3	25.3	FL81_17386	—	IPR013781 Glycoside hydrolase; catalytic domain IPR011583 Chitinase II; Missense Variant
96: 386835; 386838; 386844	1.6; 1.5; 1.3	1.7; 2.8; 3.0	FL81_16908	*C. angaria* Cang_2012_03_13_00006.g545	Synonymous Variants
102: 302190	2.3	25.6	FL81_18400	**F37C12.1**; ortholog of human *CCDC94* expressed in the pharynx, tail, and muscular, nervous and reproductive systems; predicted to encode a CWC16 protein domain.	IPR000772 Ricin B lectin domain; IPR029044 Nucleotide-diphospho-sugar transferases; Intron Variant
134: 246939	2.3	25.4	FL81_19272	**C09G5.2** *dph-2*; ortholog of human *DPH2*; predicted to have transferase activity.	IPR012885 F-box associated domain, type 2; Missense Variant
222: 31392	1.4	−1.7	FL81_20926	**F58E10.4** *aip-1*; ortholog of human *ZFAND2A* (zinc finger AN1-type containing 2A) and *ZFAND2B* (zinc finger AN1-type containing 2B); predicted to have zinc ion binding activity; involved in cellular response to misfolded protein and response to arsenic-containing substance; localizes to the cytoplasm and nucleus; expressed in the alimentary system, body wall musculature, excretory cell, head, and hypodermis.	IPR012677 Nucleotide-binding, alpha-beta plait; IPR000504 RNA recognition motif domain; Intron Variant
519: 32965	2.3	26.2	FL81_23267	**ZK550.5**; ortholog of human *PHYH* expressed in the nerve ring; human *PHYH* exhibits carboxylic acid binding activity, cofactor binding activity, and ferrous iron binding activity.	Missense Variant
1197: 1005	1.9	−1.0	FL81_24477	*C. brenneri* CBN03810, CBN11213	IPR000719 Protein kinase domain; IPR008271 Serine/threonine-protein kinase, active site; IPR002290 Serine/threonine/dual specificity protein kinase, catalytic domain; Synonymous Variant
1342: 4099; 4102	1.8	1.8	FL81_24554	**C34G6.4** *pgp-2*; predicted to have ATP binding activity and ATPase activity, coupled to transmembrane movement of substances; involved in lipid storage and organelle organization; localizes to the gut granule membrane; expressed in the Eala, Ealp, and Eara, and the alimentary and nervous systems.	IPR008250; P-type ATPase, A domain; Intron Variants

Note.—Orthologous genes in *C. elegans* and other *Caenorhabditis* species and protein domain annotations are given where available.

## Discussion

While there is strong evidence from experimental populations that completely homozygous lines can recover from fixed deleterious mutations (Burch and Chao 1999; Whitlock and Otto 1999; Estes and Lynch 2003), we find that the highly genetically diverse, outcrossing *C. remanei* did not recover from inbreeding after 300 generations in large populations. The high rate of lethality and severe fitness declines in our experiment indicate that the Ancestor was carrying many alleles that when fixed resulted in severe inbreeding depression and lethality. Our genomic data show that the Inbred line had far fewer fixations than expected under a neutral model. The high heterozygosity was distributed throughout the genome of the Inbred line. Overall, the severe reduction in fecundity with little recovery and complexity of the genomic response show that the effects of inbreeding are both severely detrimental and long lasting in *C. remanei.*

The most likely explanation based on genetic architecture for the extremely slow recovery of fitness under inbreeding—and the one most clearly supported by the genomic data—is that there are simply many more segregating deleterious mutations in natural populations. Our “ancestral” *C. remanei* population displayed high levels of polymorphism at many different sites, and over 30% of these sites were unable to be fixed during inbreeding (fig. 7). During generation of the initial inbred line, high extinction rates revealed the presence of many recessive lethal alleles or lethal combinations under close interbreeding (see also Dolgin et al. 2007; Fierst et al. 2015). Given that 99% of our inbred lines failed, this lack of fixation is most likely an outlier compared with the lethal fixation in the lines that failed.

Our results are consistent with predictions that retained heterozygosity may be due to pseudo-overdominance (Carr and Dudash 2003; Waller 2021). In the presence of many deleterious alleles linked in repulsion, excess heterozygosity would appear in regions of pseudo-overdominance, and fixation rate during inbreeding would be severely reduced (Latter 1998; Carr and Dudash 2003; Charlesworth and Willis 2009; Waller 2021). This excess heterozygosity compared with neutral expectations has been documented across diverse species: *Drosophila melanogaster* (Gilligan et al. 2005), honeybees (Smith et al. 2019), maize (McMullen et al. 2009; Brandenburg et al. 2017; Roessler et al. 2019), Eucalyptus (Hedrick et al. 2016), and rats (Eriksson et al. 1976).

Further support for maintenance of genetic diversity via pseudo-overdominance comes from our explorations of the distribution of the remaining segregating sites. We find that fixation is bimodal across the genome (fig. 5). While large regions of heterozygosity present in the Ancestor are broken up by fixation, we still see many small, punctuated spots of high heterozygosity in our inbred line (fig. 6), which have also been noted in plants (Brandenburg et al. 2017; Brown and Kelly 2020). We also see a large reduction in recombination rate across the Inbred line, indicating an increase in linkage disequilibrium potentially caused by the persistence of heterozygous haplotypes in pseudo-overdominance. Incompatibility loci that result in the maintenance of heterozygous regions have been found within the closely related selfing species *C. elegans* (Seidel et al. 2008; Bernstein et al. 2019), *C*. *tropicalis* (Ben-David et al. 2021; Noble et al. 2021) and *C*. *briggsae* (Ben-David et al. 2021). In comparison, we did not identify discrete loci or significant haplotypes in *C. remanei*. This suggests the genetic incompatibilities associated with inbreeding are complex and multilocus, similar to results found in *D. melanogaster* (Latter 1998). Further studies with more fine-scaled linkage maps or long-read data may be needed to identify and characterize regions of pseudo-overdominance. Overall, our results suggest a combination of purging, fixation, and maintenance of deleterious alleles previously segregating in the Ancestor.

### Sex Chromosome Differed from Autosomes during Inbreeding

Our genomic data showed that patterns of fixation were not consistent across the genome. The X chromosome in particular showed genetic resistance with 73% of variable sites retaining ancestral polymorphism after inbreeding. In *C. remanei*, as in other Rhabditid nematode species, females carry 2 X chromosomes (denoted XX) and males carry a single X chromosome (denoted X0) with no Y or male-specific chromosome (Nigon and Dougherty 1949; Brenner 1974). This exposes the X chromosome to different selection dynamics since recessive deleterious alleles are exposed in hemizygous condition in males and may have already been purged by purifying selection prior to inbreeding. High levels of genetic resistance on the X chromosome may imply that *C. remanei* genetic load and inbreeding resistance are related to sex-specific selection and X-autosome epistasis that differs for males and females.

### Selection during Recovery from Inbreeding

Despite the constrained recovery in fitness, we identified strong and consistent selection for alleles potentially leading to fitness recovery. We detected 102 SNPs with parallel changes across our Recovery lines. About 36 of these sites were found within 30 genes, and we were able to determine some functional information for many of these genes (table 2). The majority are involved in alimentary, muscular, nervous, and reproductive systems. The effects of the mutations with parallel recovery are variable. While 17 of the parallel recovery sites are intron variants, there were 9 synonymous variants and 7 nonsynonymous missense variants. There were two additional splice region variants. One was an intron variant, but the other potentially caused a loss of stop mutation of high impact. Given the low fitness recovery we observed and the complexity of gene interactions (Phillips 2008) these parallel changes indicate alleles with strong phenotypic effects. So, we do in fact see a clear signal for an evolutionary response, but it is spread across many different independent sites. Many, many more sites display independent response within each replicate, and many of these are likely to be functionally relevant, however, it is difficult to distinguish these from other possible effects, including genetic drift, without more formal functional validation. These genes, and the alleles we identified in the Recovery lines, are potential targets for molecular manipulation and CRISPR genome editing for studying genotype–phenotype–fitness relationships in *C. remanei*.

In contrast to our results, mutation accumulation studies have shown that it is possible to rapidly recover from complete homozygosity within experimental populations (Burch and Chao 1999; Whitlock and Otto 1999; Maisnier-Patin et al. 2002; Estes and Lynch 2003). Back-mutations at deleterious sites and beneficial mutations are thought to be rare (Smith 1978), but compensatory mutations may counteract fixed deleterious alleles and aid in fitness recovery (Burch and Chao 1999; Whitlock and Otto 1999; Maisnier-Patin et al. 2002; Estes and Lynch 2003). Mutation accumulation and recovery studies in *C. elegans* have demonstrated similar processes with compensatory epistatic mutations swept to fixation during recovery (Estes and Lynch 2003; Denver et al. 2010; Estes et al. 2011). For example, in a *C. elegans* mutation accumulation experiment 28 new mutations occurred and rose to fixation within 10–20 generations (Denver et al. 2010). Many of the new mutations had predicted interactions with well-characterized loci that had fixed during mutation accumulation, suggesting that these new mutations had compensatory beneficial effects.

Our results stand in stark contrast with these previous studies. There are several possible explanations for the difference in our results. First, it is possible that the landscape for compensatory mutations might differ across the species. While this seems extremely unlikely, it is a formal possibility that our data cannot directly address. More likely is a difference in how compensatory mutations interact with differences in mating systems between *C. elegans* and *C. remanei.* Under self-fertilization in *C. elegans*, compensatory mutations that arise in a given genetic background, even if they are on a different chromosome, are very likely to be inherited with the target deleterious mutation because, although recombination does occur, it has little effect on genetic diversity when the rest of the genome is nearly completely homozygous. In contrast, obligate outcrossing in *C. remanei* increases the effectiveness of recombination in breaking up different genetic combinations, especially in large populations. This may make it more difficult for epistatically interacting loci to remain together on the same genetic background (Phillips 2008). On the other hand, in *C. elegans* other deleterious mutations that are not “fixed” by the compensatory mutation are locked in the genome, whereas in *C. remanei*, different combinations of adaptive mutations can recombine into a common background much more easily, which should be relevant on the timescales of this study. More importantly, since our experiments were initiated from a highly inbred state, recombination would have little impact on changing the dynamics of deleterious mutations that are already fixed in the population, since they would be present on every genetic background upon which a new compensatory mutation might find itself. Overall, then, while differences in mating systems in species used in mutation accumulation and our genetic recovery experiments could explain some of the differences in results, they are unlikely to explain the extreme difference in rate of total fitness recovery across approaches. Recent simulation work shows that pseudo-overdominance has a negative effect on selection, and could therefore contribute to lack of recovery (Sianta et al. 2021).

### Deleterious Mutations and Aging

Unlike fecundity, lifespan did not show any decrease under inbreeding. Instead, the Recovery lines evolved an increase in lifespan when compared with both the Ancestor and Inbred (fig. 2*B*). The basic premise of inbreeding depression is traits decline in value because deleterious alleles will always have a negative effect on traits under positive directional selection. A lack of decline in longevity with inbreeding would therefore suggest that longevity itself is not under selection, nor is it strongly correlated with other traits under selection. This result is consistent with an experimental evolution study in *C. elegans* which did not find any evidence for a tradeoff between early reproduction and longevity (Anderson et al. 2011). Alternatively, the alleles involved in lifespan extension could have been physically or statistically linked to a region under selection in the Recovery lines. We did identify parallel allelic changes in FL81_06442, a *C. remanei* protein orthologous to the *C. elegans* protein R05A10.2. This protein is affected by *daf-2*, an aging factor, in *C. elegans* (Kenyon et al. 1993) and may be a target for further studies investigating lifespan in *C. remanei*.

### Genetic Basis of Inbreeding Depression

Despite some hopeful indications based on earlier mutation-accumulation studies, our results indicate that species with high genetic load may not be capable of recovery from inbreeding. For the nematode *C. remanei*, severe inbreeding depression is almost certainly caused by the very large number of segregating deleterious alleles in the population prior to inbreeding. Part of the complexity of the genetic basis of inbreeding depression in this species is due to the very large effective population sizes at which it exists in nature. It is possible that species with smaller population sizes might have few segregating alleles before inbreeding, leading to less severe fitness effects. On the other hand, those species are also unlikely to exist at large enough population sizes to allow a sufficient number of mutations to enter the population before demographic factors drive the population to extinction. Overall, our results suggest that evolution is unlikely to lead to rapid rescue of endangered populations, at least from a genetic point of view.

## Materials and Methods

### Laboratory Culture

Prior to experiments, worm strains were maintained using standard protocols on Nematode Growth Medium-lite (NGM-lite; US Biological, cat. N1005), with *Escherichiacoli* strain OP50-1 at 20 °C (Brenner 1974).

### Inbreeding

To overcome the extinction reported for *C. remanei* (Dolgin et al. 2007) a novel scheme was used to create inbred line PX356 from ancestral strain EM464 (Fierst et al. 2015). *C*. *remanei* EM464 (formerly *C*. *vulgaris BK*) culture was originally isolated in New York City, established from a single gravid female, and obtained from the Caenorhabditis Genetics Center, University of Minnesota, Minneapolis, MN (Baird et al. 1994; Sudhaus and Kiontke 1996). Two hundred independent lines of Ancestor were subjected to brother-sister mating with just 2 lines remaining at generation 7. These lines were crossed and maintained for 20 generations as a single outcrossing population. From this population 100 lines were subjected to brother-sister mating for 23 generations until only one surviving Inbred line, PX356, remained (fig. 1; Fierst et al. 2015). Worms were not allowed to starve during inbreeding or experimental treatments.

### Maintenance of Recovery Lines

The Inbred line was denoted Generation 0 and three Recovery lines were independently established from this line and maintained on standard petri dishes of NGM-lite agar seeded with OP50-1 at 20 °C. Recovery lines were propagated by transferring a piece of agar from a populated petri dish and placing it upside down on the agar surface of a new petri dish every 3–4 days. Petri dishes were checked under the microscope to ensure that the removed piece contained large numbers of individuals (usually several hundred). Each transfer event was counted as one generation. Populations grew to census sizes of >2,000 individuals in-between transfers. Every 20 generations, lines were frozen and stored.

### Experimental Setup

After experimental inbreeding and recovery, fecundity and longevity assays were conducted on the previously frozen population samples. Lines were recovered by thawing a tube of frozen worm solution at room temperature and pipetting 1 ml of worm solution onto an agar plate. The Inbred line was included in each experiment to serve as the control. After lines were maintained at 20 °C for at least three generations, individuals were age-synchronized by bleaching. Lines were rinsed into 15 ml conical tubes and resuspended in 5 ml of S. basal. Then, 300 µl of 4M NaOH followed by 600 µl of 6% sodium hypochlorite were pipetted into the worm solution. The worm/bleach mixture was then shaken for 3 min and centrifuged at high speed for 2 min. The supernatant was discarded and the worms were resuspended to 10 ml with S. basal. This step was repeated 3 times or until the odor of bleach was undetectable. The remaining embryos were resuspended to a final volume of 7 ml in S. basal and placed on a rotator overnight at 20 °C. The following day, the solution of L1s was pipetted onto the edge of a 100 mm agar plate and stored at 20 °C. Synchronized populations were stored at 20 °C for approximately 48 h until the worms reached the fourth larval stage.

### Fecundity Assays

For each line, 40 replicates of 35 mm agar plates containing 1 virgin L4 female and 3 L4 males were stored at 20°C. Plates were randomized in order to prevent bias.

Every 24 h for 1 week, the worms were transferred to new 35 mm agar plates. If males died, they were replaced from the corresponding population plate to maintain three males per one female at all times. *C*. *remanei* are sperm-limited in their reproduction (Palopoli et al. 2015) and this ensured sufficient sperm for fecundity measures. The plates the worms were transferred from were kept at 20 °C for 2 days, after which L4 progeny were counted and female deaths recorded. Any females that desiccated on the plastic wall of the petri dish before the end of 1 week were censored from the final data set. Any females that died of other causes within 3 days were also censored from the final data set. Lines were compared with paired t tests with Bonferroni corrections.

### Longevity Assays

For each line, 30 replicates of 35 mm plates containing 5 virgin L4 females were stored at 20 °C. Plates were randomized in order to prevent bias and examined every 1–2 days to check for dead individuals. If an individual was not moving, the plate was tapped on the counter. If no movement occurred after this stimulus, the tail of the worm was nudged using a platinum wire. If no movement occurred after this stimulus, the head of the worm was nudged using a platinum wire. If no movement occurred after applying all stimuli, the individual was recorded as dead and its position on the plate marked by a red circle. Each day, any carcasses circled the previous day would be subjected to the aforementioned stimuli in order to ensure that the death was correctly determined. Individuals that desiccated on the plastic wall of the petri dish were censored from the final data set. Individuals were transferred to new petri dishes on Day 10 of the experiment and every 7 days after that to ensure adequate amounts of the bacterial food source and to avoid contamination.

### DNA Isolation

DNA was isolated from pooled population samples (1,000 s of worms washed from plates) and sequenced on an Illumina HiSeq instrument. Recovery Lines 1–3 were sequenced as single end DNA reads after 100 generations and Recovery Line 2 was sequenced as single end DNA reads after 200 generations. Recovery Lines 1 and 3 were sequenced as paired end DNA reads after 200 generations. The Recovery Lines still had very low fitness recovery at 300 generations and we made the decision to not proceed with whole genome sequencing.

### Genetic Analyses

DNA libraries were aligned to the PX356 reference sequence NMWX00000000.1 using two alignment tools: BWA mem (Li and Durbin 2009) and GMAP-GSNAP (Wu et al. 2016). The BWA alignment was used for all analyses, except for allele frequency estimation step where we required each site to be considered an allele by MAPGD in both alignments (Lynch et al. 2014; Ackerman et al. 2017). Picard Tools (Broad Institute 2016) and the Genome Analysis Toolkit were used to mark duplicates, realign indels, and filter noise in alignment (McKenna et al. 2010; DePristo et al. 2011).

Allele frequencies were estimated with the *pool* function in the MAPGD software package (version 0.4.33; Lynch et al. 2014; Ackerman et al. 2017). Sites with missing data were removed and SNPs with a log-likelihood ratio >22 and a minor allele frequency >5% were considered to be true segregating variants. We required segregating sites to meet these criteria for both BWA (Li and Durbin 2009) and GSNAP (Wu et al. 2016) alignments to reduce false positives and remove sites with ambiguous alignment (Kofler et al. 2016) and used the BWA allele frequencies in analyses. Tri-allelic sites are also removed by MAPGD.

Because our data were a somewhat heterogeneous combination of paired end and single end sequences at different read depths, we sought to remove potential biases. In particular, segregating polymorphisms were increased in both paired end and high depth samples (supplementary table 2, Supplementary Material online), and we removed sites with segregating variants in paired-end sequences that displayed fixation (no polymorphism) in the single-end samples. These sites may have been true polymorphisms, but with our design they could not be distinguished from sampling error.

Sites were filtered for coverage (all bioinformatics scripts and workflows are available at https://github.com/BamaComputationalBiology/Inbreeding). Inbred was sequenced at a high mean read depth of 370× while Ancestor and Recovery were sequenced to mean depths of 25–64× (supplementary table 2, Supplementary Material online). Ancestor and Recovery lines were filtered for a minimum sequence read coverage per site of 5×, while the Inbred line had a minimum coverage cutoff of 10% (37× coverage) of the mean coverage per site for the Inbred line. The maximum coverage was 3× the mean coverage for all lines according to Li (2014). The hard-masked genome fasta was downloaded from wormbase (https://parasite.wormbase.org/Caenorhabditis_remanei_prjna248909/Info/Index/), and converted to a bed file format using a script from Cook (2019). These ranges were then used to hard mask the variants with BEDTools (Quinlan and Hall 2010).

After rigorous filtering we were left with 150,348 (0.13% of the 118.5 Mb assembled genome) segregating variants. We calculated the site frequency spectrum (SFS) for each sample using the minor allele frequencies at each variable site (Fisher 1931; Wright 1938).

To calculate expected heterozygosity after 23 generations of full-sib inbreeding we used the formula ∼1.17 *h_0_*(0.809)^*t*^ where *h_0_* is initial heterozygosity and *t* is the number of generations (Nagylaki 1992). It is difficult to exactly calculate a neutral expectation for homozygosity under our inbreeding design because the brother-sister mating was paused at generation 7 and then continued for an additional 23 generations (fig. 1). However, we can use 23 generations of inbreeding as a minimum for our homozygosity expectation, noting that the true expectation will be somewhere between 23 and 30 generations of inbreeding.

### F_ST_

We used the software package PoolFstat (Hivert et al. 2018) to calculate the fixation index (F_ST_) between population pairs for each variable SNP. We calculated the mean F_ST_ for each gene by averaging across variant sites 1 kb upstream of the gene, within the gene and 1 kb downstream of the gene.

### Distribution of Heterozygosity

To look at genome-wide distribution of heterozygosity, we averaged minor allele frequencies across 1 kb windows for each population. We defined a fixed region as a contiguous portion of the genome 1 kb or greater in length where minor allele frequency did not exceed 3%. This was roughly the threshold of detection (equivalent to 1–2 sequence reads) for our samples that were sequenced as single end reads. This procedure eliminates small, fixed regions and may underestimate the size of fixed regions. We chose to take this approach to focus on genome-wide patterns for which we had rigorous support.

### Allele Frequency Trajectories

We separated sites by allele frequency trajectories to identify the major trends occurring during inbreeding and recovery. Due to the error rates and uncertainty in pooled sequencing, the cutoff used for “fixation” was 5% minor allele frequency, the same allele frequency that was used to call variable sites. Any minor allele under 5% was considered fixed out of an abundance of caution (Li 2014). “Fixation” sites were defined as segregating in the Ancestor and <5% minor allele frequency in all Inbred and Recovery lines. “Intermediate” sites were those that remained segregating in the Ancestor and Inbred line whose allele frequency changed <50% through inbreeding and recovery. The remaining sites were filtered into two trends: 1) “Bounce” sites had low frequency in the Ancestor, higher frequency in the Inbred, and lower frequency in recovery; and 2) “Up” sites increased in frequency during both inbreeding and recovery. These categories allow us to characterize what proportion of variable sites were fixed through inbreeding and, of the remaining sites, how segregating variation changed through recovery.

### Recombination Rates

Recombination rates across the samples were calculated with the ReLERNN (version 1.0.0) software pool-sequencing pipeline with default settings (Adrion et al. 2020). ReLERNN required more sites than our highly filtered data set of 150,348. So we used the MAPGD allele frequency calls for the BWA alignment in the Inbred and Ancestral line without the MAPGD’s log-likelihood cut off. We applied only the coverage filter described in the “Allele Frequency Estimation” section above, and then filtered out sites where the sample was fixed because ReLEARN runs on variants in an individual pooled sample. This resulted in 5,557,381 variable sites for the Inbred line, and 1,495,382 sites for the Ancestral line. ReLERRN was then ran separately on the ancestral and inbred line using the pooled-sequencing pipeline with default settings and the hard-masked regions from the “Genetic Analyses” section. ReLERRN outputs the recombination rate within a window selected by the program. We deleted any windows where the recombination rate was 0, caused by poor coverage in those windows. We did not have adequate sampling in the Ancestor to look at variation across the linkage blocks; however, we were able to average across the linkage block to get an estimate of the recombination rate on that contig.

The Haplovalidate software package (version 0.1.6) was used to identify potential haplotype blocks in the inbred and recovery samples (Franssen et al. 2017; Otte and Schlotterer 2021). We performed a CMH test to analyze parallel changes in allele frequencies between the Inbred and Recovery lines at generation 100 and 200 with the software package PoPoolation2 (Kofler et al. 2011), which was then provided to Haplovalidate along with allele frequencies. Sites were filtered for significant changes in the recovery lines (*P*-value < 0.05). Haplovalidate parameters were set to “wins=seq(0.1,10,0.05), mncs = 0.03,” and the settings for running Haplovalidate were “takerandom = 2000, filterrange = 5000, findthreshold = 5.”

### Effective Population Size

Effective population sizes were calculated with the software package PoolSeq (Taus et al. 2017). In order to test the influence of parameters on effective population size estimation, we used two Census sizes and two “Poolsizes” (supplementary table 3, Supplementary Material online). For the census size we used 1,000,000 (the estimated effective population size for the species; Cutter et al. 2006) and 1,500 (the approximate population size of a plate of nematodes). For PoolSize (size of the pool sequenced together) we tested with 1,500 and 500. PoolSeq uses allele frequencies across replicated, temporal, pool-sequencing data to estimate genome-wide effective population size (N_e_) in each sample described in Jonas et al. (2016) and Taus et al. (2017).

### Selection Scans in Recovery Lines

In order to test for parallel changes in allele frequency in the Recovery lines, we fit a GLM with quasibinomial error distribution to the allele frequency changes across the Inbred line, generation 100 Recovery, and generation 200 Recovery according to the Wiberg et al. (2017) recommendation for best practices with pooled sequencing data. All sites that were significant in the quasibinomial-GLM analyses were also significant with the CMH test (see section above: Recombination Rates). We used the R software package *qvalue* for false discovery rate correction (Storey et al. 2019). Sites with significant changes (i.e., quasibinomial-GLM q value < 0.05) across all three Recovery lines were associated with genic or intergenic locations with BEDTools (Quinlan and Hall 2010). Proteins containing significant SNPs were annotated for putative molecular functions with the Interproscan software package (Jones et al. 2014) and orthologous genes in other *Caenorhabditis* species identified with OrthoFinder (Emms and Kelly 2015). We searched WormBase ParaSite for functional information for orthologous genes (Howe et al. 2017). We also used snpEff to characterize the effects of the significant SNPs within genes (Cingolani et al. 2012).

## Supplementary Material

Supplementary data are available at *Molecular Biology and Evolution* online.

## Supplementary Material

msab330_Supplementary_Data
