# Supplementary material for: Slow Recovery from Inbreeding Depression Generated by the Complex Genetic Architecture of Segregating Deleterious Mutations
Source: Mol Biol Evol. 2021 Nov 17;39(1):msab330. doi: 10.1093/molbev/msab330 (PMC8789292; doi:10.1093/molbev/msab330)
Supplement: msab330_Supplementary_Data [file msab330_supplementary_data.docx]

Slow recovery from inbreeding depression generated by the complex genetic architecture of segregating deleterious mutations:

**Supplementary Materials**

Paula E. Adams^1^, Anna L. Crist^2^, Ellen M. Young^3^, John H. Willis^3^, Patrick C. Phillips^3^, Janna L. Fierst^1,4*^

^1^ Department of Biological Sciences, University of Alabama, Tuscaloosa, AL, USA

^2^ Department of Virology, Institut Pasteur, Paris, France

^3^ Institute of Ecology and Evolution, University of Oregon, Eugene, OR, USA

^4^ Current Address: Department of Biological Sciences, Florida International University, Miami, FL, USA

*Author for correspondence: Janna Fierst: jfierst@fiu.edu

**Supplementary Table 1.** Number of observations, mean, and standard deviation for fecundity per individual.

| Line | No. of Observations | Mean | Standard Deviation |
| --- | --- | --- | --- |
| Ancestor | 78 | 493.6410256 | 320.818109 |
| Inbred | 153 | 181.7581699 | 103.1142076 |
| Gen100 | 120 | 223.525 | 126.5079107 |
| Gen200 | 155 | 244.4387097 | 144.6610047 |
| Gen300 | 120 | 206.7833333 | 129.0708348 |

**Supplementary** **Table 2.** Coverage, standard deviation, minimum filter coverage, and maximum filter coverage for each line. Coverage filters were applied to MAPGD output. Also shows total SNPs per sample after filtering.

| Line | Mean Coverage | Standard Deviation | Minimum Coverage Cutoff | Maximum Coverage (3*mean) |
| --- | --- | --- | --- | --- |
| Inbred | 375.51 | 245.07 | 37 | 1127 |
| Ancestor | 24.82 | 11.75 | 5 | 75 |
| Recovery 1, Generation 100 | 22.62 | 10.86 | 5 | 68 |
| Recovery 1, Generation 200 | 64.40 | 68.69 | 5 | 194 |
| Recovery 2, Generation 100 | 26.22 | 11.12 | 5 | 79 |
| Recovery 2, Generation 200 | 23.48 | 10.63 | 5 | 71 |
| Recovery 3, Generation 100 | 23.05 | 10.22 | 5 | 70 |
| Recovery 3, Generation 200 | 39.15 | 44.85 | 5 | 118 |

**Supplementary Table 3.** Effective population sizes for the Inbred and Recovery lines.

| **Census: 1,000,000, PoolSize=1500** | **Inbred Population Size Estimated from Ancestral State** | **100 Generation Population Size Estimated from Ancestral State** | **200 Generation Population Size Estimated from Ancestral State** |
| --- | --- | --- | --- |
| Rep1 (NYLT_2) | 28.23 | 90.28 | 144.56 |
| Rep2 (NYLT_3) |  | 90.06 | 150.9 |
| Rep3 (NYLT_5) |  | 90.62 | 147.23 |
|  | **28.23** | **(mean) 90.32** | **(mean) 147.5633333** |
|  |  |  |  |
| **Census: 1,500, PoolSize=1500** | **Inbred Population Size Estimated from Ancestral State** | **100 Generation Population Size Estimated from Ancestral State** | **200 Generation Population Size Estimated from Ancestral State** |
| Rep1 (NYLT_2) | 28.19 | 90.16 | 144.38 |
| Rep2 (NYLT_3) |  | 89.94 | 150.7 |
| Rep3 (NYLT_5) |  | 90.5 | 147.04 |
|  | **28.19** | **(mean) 90.2** | **(mean) 147.3733333** |
|  |  |  |  |
|  |  |  |  |
| **Census: 1,500, PoolSize=500** | **Inbred Population Size Estimated from Ancestral State** | **100 Generation Population Size Estimated from Ancestral State** | **200 Generation Population Size Estimated from Ancestral State** |
| Rep1 (NYLT_2) | 28.27 | 90.4 | 144.75 |
| Rep2 (NYLT_3) |  | 90.18 | 151.1 |
| Rep3 (NYLT_5) |  | 90.74 | 147.42 |
|  | **28.27** | **(mean) 90.44** | **(mean) 147.7566667** |

**Supplementary Figure 1.** The mean and median ROH increased from the Ancestor to Inbred lines. The figure above shows the distribution of ROH by size for the Ancestor and Inbred lines with quartile lines in horizontal black within the distributions.


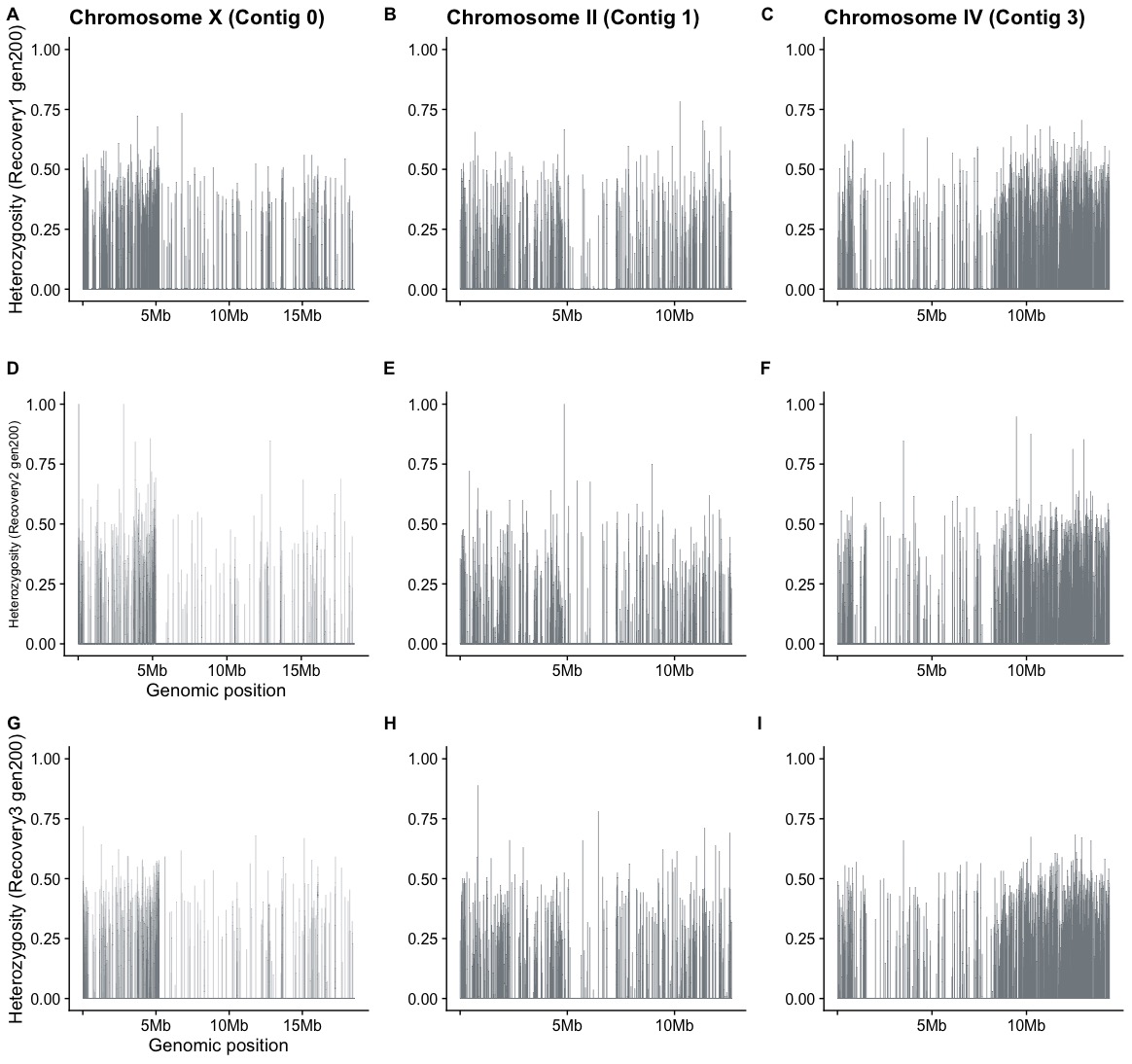


**Supplementary Figure 2.**

Average heterozygosity in 1kb blocks across the 3 largest linkage groups (corresponding to (A) Chromosomes X, (B) II and (C) IV) show that polymorphism in the Recovery lines was similar to the Inbred Line. Residual segregating polymorphisms are not evenly distributed along chromsomes and there are distinct regions of Chromosome X and IV that retain polymorphisms.
